# Experience and trust: the benefits of mate familiarity are realized through sex-specific specialization of parental roles in Cassin’s auklet

**DOI:** 10.1098/rsos.241258

**Published:** 2024-12-18

**Authors:** Amy Yanagitsuru, Christopher Tyson, Frédéric Angelier, Michael Johns, Thomas Hahn, John Wingfield, Haley Land-Miller, Rebecca Forney, Elisha Hull

**Affiliations:** ^1^ Department of Neurobiology, Physiology, and Behavior, University of California, Davis, CA, USA; ^2^ Department of Animal Science, University of California, Davis, CA, USA; ^3^ Centre d’Etudes Biologiques de Chizé, CNRS, La Rochelle Université, Villiers en Bois 79360, France; ^4^ Point Blue Conservation Science, Petaluma, CA, USA

**Keywords:** parental behaviour, monogamy, sex difference, seabird

## Abstract

Maintaining a pair bond year after year (perennial monogamy) often enhances reproductive success, but what familiar pairs are doing differently to improve success is unknown. We tested the hypothesis that endocrine changes mediate improvements in parental attendance in known-age Cassin’s auklets *Ptychoramphus aleuticus*, for which we found limited evidence. Instead, we found sex-specific parental roles in familiar pairs. Males modulated their nest attendance depending on the attendance of their mate, but the direction depended on mate familiarity. We suggest his flexibility may be mediated by prolactin. In a historical dataset, females with a familiar mate laid larger eggs that hatched into more robust chicks, but larger eggs correlated with lower female body condition. In study birds, attendance by males and females in good condition predicted chick weight, but attendance by females in poor condition did not, suggesting female-specific energetic constraint. Our findings suggest that males and females contribute differently to their joint reproductive fortunes, and that improvements in their respective roles may result in the benefits of mate familiarity. Since improved reproductive success is presumed to be a main benefit of maintaining a long-term pair bond, these results suggest a new avenue of research in the evolution of monogamy.

## 1. Introduction

Monogamy is a rare breeding strategy in animals, although it is found in a few species spread across many different taxonomic groups. The exception is birds, among which it is very common, and it is close to universal in seabirds [1–3]. The evolution of monogamy and its prevalence in birds has received significant scientific interest, but there remain many unresolved questions about how monogamy evolves and how it is maintained. Many explanations for the evolution of monogamy have been posed, such as being a consequence of mate guarding and territoriality [4,5] or a strategy for avoiding infanticide [6] or enhancing parental care [7]. The latter is of particular interest, as parental care by two parents (biparental) and monogamy often occur together. Biparental care probably plays a role in the maintenance of monogamy on an evolutionary scale, since offspring raised by two parents generally mature in better condition than those raised by single parents [8–10].

Many seabirds exhibit perennial monogamy—an extreme form of social monogamy where individuals breed with the same mate year after year [3]. A hypothesized benefit of perennial monogamy is the ‘mate familiarity effect’, where reproductive success improves when breeding with a familiar mate. This effect has been observed in a variety of taxa [11–14], including seabirds [15–20], and represents a potent evolutionary mechanism by which monogamous mating strategies are maintained. Despite its prevalence, what experienced and inexperienced pairs are doing differently that results in improved reproductive success through the mate familiarity effect has been difficult to identify.

Understanding the causes and consequences of perennial monogamy can foster understanding of what evolutionary forces support long-term reproductive partnerships in vertebrates. Our aim was to contribute to this goal by evaluating the endocrine and behavioural mechanisms that underly the mate familiarity effect in the context of parental behaviour. Endocrine control of parental behaviour is well-established, so hormones provide a window into physiological processes that would otherwise be difficult to measure in a free-living wild animal [21–23]. We hypothesized that the benefits of mate familiarity are due to endocrine-associated behavioural shifts when breeding with a familiar mate that produce enhanced reproductive success.

While there are demonstrated benefits of mate familiarity in the form of earlier breeding [24,25], possibly through less time spent courting or establishing a nest site, mate familiarity probably benefits pairs after the egg has been laid as well. For example, better coordination in nest attendance, which may improve with a longer pair bond, improves reproductive success [26,27]. We focused our study on the parental phases of breeding, which we define as incubation and chick rearing. There are multiple endocrinological mechanisms that may play a role in the mate familiarity effect, but during the parental phases, two of the most compelling hormones are prolactin (PRL) and corticosterone (CORT).

PRL is an anterior pituitary hormone highly linked to parental behaviour in a variety of taxa [28], including birds [29,30]. Elevation in PRL is associated with increased parental behaviours [31–33] and reduction with decreased parental care and, in some cases, abandonment [34–36]. More years of previous breeding by an individual also elevates PRL, which could translate to increased parental investment and improved reproductive performance [37–39]. This result is often found in seabirds [40–42]. Corticosterone is a steroid hormone highly correlated with both acute and chronic stressors [43] in addition to an important role in metabolism [44], and is thought to play a role in promoting survival under adverse conditions [45] by modulating metabolism, escape behaviour, immune function and other physiological and behavioural adaptations [43]. In reproduction, CORT is elevated when breeding with an unfamiliar mate [46] or for the first time [41,47]. It also has implications for reproductive success: elevated CORT is linked to reduced PRL and parental care [48] and abandonment [49,50]. The interaction between these two hormones has been hypothesized to mediate parental decision making [21], but few, if any, studies have investigated the behavioural effects of the relationship between PRL and CORT in the context of mate familiarity.

To integrate physiology, behaviour and fitness in the context of the mate familiarity effect, we used the Cassin’s auklet *Ptychoramphus aleuticus* as a model system during incubation and chick rearing across 2 years. Cassin’s auklets are small, planktivorous seabirds that form long-term pair bonds of up to 12 years and show evidence of the mate familiarity effect, with improved hatching success of their single egg with a longer pair bond [51,52] and greater lifetime reproductive success with greater lifetime mate fidelity [53] from the time they begin breeding at age 2 or 3. In the population used for this study, we have observed highly variable mate fidelity, with some individuals breeding with a new mate frequently while others stay with the same mate until one of the two individuals disappears from the population. This variation is extremely useful in disentangling the effect of pair experience from the effect of age and individual experience.

During incubation, auklet parents alternate shifts, with one parent incubating while the other feeds at sea. Switches occur at night and birds are seldom encountered outside the burrow during the day [54]. While Cassin’s auklet eggs can tolerate some neglect if the incubating parent leaves before the feeding parent returns, neglect prolongs development [55]. During chick rearing, both parents return once nightly to feed the chick [54]. The attendance of both parents during both incubation and chick rearing is necessary to raise the chick to fledging. In our experience, when one parent died or abandoned the nest, the remaining parent was unable to raise the chick alone. Because of the importance of nightly attendance by both parents, our measure of parental behaviour was nest attendance rates.

Our study objectives were as follows. *Objective 1*: identify the endocrine predictors of nest attendance rate in males and females across a range of mate familiarity and age by measuring PRL and CORT, with the prediction that elevated PRL and low CORT would correlate with increased attendance, and that experienced pairs would show high PRL, low CORT and high attendance more often than inexperienced pairs. *Objective 2*: identify physiological and behavioural predictors of reproductive performance by measuring hatching success and chick weight in all study pairs, with the prediction that elevated PRL, low CORT and high attendance would be correlated with higher hatch probability and chick weight and that both hatch probability and chick weight would be elevated in experienced pairs. *Objective 3*: identify predictors of egg volume using historical data, with the prediction that larger egg volumes would be correlated with lower female body condition. If a larger egg comes with a cost to her body condition, we further predicted that larger eggs would be more likely to hatch, and hatch into chicks that reached a larger maximum size and were more likely to fledge. Objective 3 was added post hoc, in response to sex differences we observed in the results for objectives 1 and 2, and consequently was evaluated using previously collected data from different individuals than objectives 1 and 2.

## 2. Material and methods

### 2.1. Fieldwork and sample collection

We conducted our study on Southeast Farallon Island (37.6989° N, 123.0034° W; Farallon Islands National Wildlife Refuge, CA, USA) in 2019 and 2021. These 2 years represented a positive El Niño Southern Oscillation (ENSO) event where on average only 8% of followed pairs fledged a chick (2019), and a negative ENSO event (2021) where 65% of pairs fledged a chick [56]. Many individuals of the Southeast Farallon Island Cassin’s auklet population are of known age and have known reproductive histories [53], allowing us to disentangle the confounding effects of age and mate familiarity. Nest boxes were checked every 15 days until an incubating bird was found. If the individual was of known age, it was measured and evaluated for inclusion in the study. All morphometrics are therefore from birds in their first two weeks of incubation. We weighed known-age incubating adults between 13.00 and 15.00 Pacific Standard Time (PST) with a Pesola spring scale (±1 g), measured wing cord with a wing rule (±1 mm) and measured bill depth with a digital calliper (±0.1 mm). The following day, we measured the mate. We checked nests every 5 days to determine egg fate, and after hatching, chicks were weighed every 5 days until fledging to measure the maximum weight attained.

We acknowledge that sex is a non-binary phenotype and that treating it as such can obscure important and relevant biological information, and inadvertently support social agendas that cause real harm [57]. For brevity and clarity, we refer to the egg-laying member of the pair as ‘female’ and the non-egg laying member of the pair as ‘male’. Males have deeper bill depths compared with females, but there is significant overlap [58]. To sex birds accurately, we used bill depth-based sex estimations from multiple previous years for all individuals that had been measured before with a different mate (63 of 76 individuals in 2019 and 66 of 80 individuals in 2021), assuming a pair was one male and one female. Sex-specific body condition was estimated using the residual of the model mass ~ bill depth + wing cord. This model was built using morphometrics for this colony from 1973 to 2021 (*n* = 7964).

Pairs were selected for the study if there was at least one known-age mate. We attempted even representation of individuals in their first or second breeding attempt (i.e. 2–3 years old; ‘young’; in this population many individuals breed for the first time at 2 years) or had more years of previous breeding (i.e. 5+ years old; ‘old’). If the pair contained an individual of unknown age, the pair was classified based on the known-age mate, but because ages of mates are only weakly correlated (coefficient = 0.39, s.e. = 0.051, *p* < 0.001, *R*^2^ = 0.14, *n* = 353), we used the categorical classification of pairs as ‘old’ or ‘young’ for pair selection purposes only. We used individual age for 97 known-age birds and band year-based age estimation for 51 non-known-age mates of known-age birds in our models. Although there were nine individuals mistakenly classified because they were not thought to be known age and were later discovered to have been banded as chicks, there was no difference in the mean age between experience groups (Student’s *t*-test, *p* = 0.3). When we compared newly formed pairs (‘inexperienced’) with pairs that had been observed breeding together at least once before (‘experienced’) in our analyses, we used a categorical variable to describe mate familiarity (i.e. ‘experienced’ or ‘inexperienced’; ‘pair experience’). When exploring changes over time, we used the number of years the pair had been observed breeding together (‘bond duration’). In this population, known-age individuals are usually recaptured yearly from age 2 or 3 until senescence, so it is unusual to have gaps in our knowledge of their reproductive history. We followed 79 auklet pairs over 2 years, 19 inexperienced pairs and 20 experienced pairs in 2019 and 19 experienced and 21 inexperienced pairs in 2021. A total of 145 individuals were used in this study, 23 of which contributed to the study in both years.

To measure nest attendance, nest boxes were fitted with radio frequency identification (RFID) readers and both individuals were fitted with a passive integrated transponder (PIT) tag on the tarsus, a method that is reliable for determining nest attendance and causes little disturbance [59,60]. Our RFID readers were programmed to continuously record from two circular antennas deployed at the nest entrance to record tagged birds entering or exiting the nest box. Tags and readers were deployed 1–2 days after birds were weighed and measured. We measured nest attendance rate as the proportion of nights an individual was present at the nest. During incubation, presence at night means the bird was either finishing an incubation bout or relieving its mate. During chick rearing, presence at the nest at night was assumed to be prey delivery. Every effort was made to equalize the number of nights of recordings across nests, but it was not always possible as readers occasionally ran out of battery or malfunctioned. We accounted for this variation in our statistical analysis by weighting models by the number of nights of RFID recording.

Physiological sampling was conducted 10 days after the egg was first observed during incubation (13–16 May 2019 and 5–17 April 2021) between 10.00 and 19.00 PST and when the chick was 10 days old (17–24 June 2019 and 28 April–19 May 2021) between 22.00 and 0.00 PST, as Cassin’s auklets are only present on the island at night during chick rearing. A one-time measurement will not fully capture the range of hormones across a breeding period, which can be inconsistent within an individual [61]. However, repeated sampling would cause levels of disturbance that nesting auklets may not tolerate and we were unwilling to risk triggering nest abandonment, given that this population has significantly declined since monitoring began in the 1970s [53]. We therefore limited our disturbance of the colony, at the cost of a lower likelihood of finding correlations between behaviour and hormone levels. We measured PRL in plasma and CORT in faeces, since faecal CORT is less susceptible to momentary stressors from investigator disturbance [62]. An analysis of faecal CORT data collected in this population in 2021 show no correlation between CORT and time of collection (generalized linear mixed model with faecal sample emulsification as a random effect, *p* = 0.61). To our knowledge, there is no existing assay for measuring PRL in avian faeces, but it takes much longer than CORT to respond to handling stress [63]. Other studies have found little evidence of circadian rhythm in plasma PRL [64]. We collected a faecal sample by holding the bird over a Pyrex container for no more than one minute, then collected a 150 µl blood sample from the brachial vein into heparinized capillary tubes to measure PRL. Plasma and faecal pellets were frozen at −10°C in the field. After the field season, plasma samples were frozen at −80°C and faecal samples at −15°C.

### 2.2. Laboratory methods

We dried faecal samples in a fume hood, then weighed and homogenized them before rehydrating with 2 ml dH_2_O. To validate our radioimmunoassay protocol, we made a pool from three samples, added 1 and 0.25 ng CORT and performed serial dilution to confirm parallelism to the standard curve. We measured 1.05 and 0.15 ng CORT in these pooled samples. Steroids were extracted in duplicate following Krause *et al*. [65] across two assays. Mean per cent recovery was 61.5% (s.d. = 20.6%). Intra-assay CV was 3.66% and inter-assay CV was 7.16%. Twenty-six out of 194 faecal samples were emulsified by dichloromethane during steroid extraction, which decreased the resulting CORT measurement by 6.8 ng mg^−1^ (s.e. = 1.2, *t*-value = 8.70, *p* < 0.001). There was no evidence that emulsified samples were non-randomly distributed among any variables of interest (electronic supplementary material, table S1).

We determined plasma concentrations of PRL through heterologous radioimmunoassay as detailed in Cherel *et al*. [66] in 20 µl duplicates across two assays. The detection limit was 0.99 ng ml^−1^. The intra- and inter-assay CVs were 11.20% and 12.56%, respectively. Pooled plasma samples of Cassin’s auklet produced a dose–response curve that paralleled chicken prolactin standard curves, validating our PRL radioimmunoassay for this species.

### 2.3. Historical data

We used historical data of known-age Cassin’s auklet egg length and diameter from Southeast Farallon Island (the same population as our study birds) from 1992 to 2017 (*n* = 783). Egg length and diameter of the widest point were measured using digital callipers (±0.1 mm) at the same time as adults were measured in early incubation. Egg measurements of the study birds were not available because objective 3 was added post hoc, in response to interesting patterns we observed in our results for the first two objectives. Accordingly, egg measurements were not in our original study design and regular egg measurement was discontinued from the monitoring protocol in 2018. We calculated egg volume as 0.5202 *LD*^2^ − 0.4065 [67], where *L* is the length and *D* is the diameter. These data were paired with information on pair experience, female age, wing cord (a proxy of body size), body condition, hatching success and the maximum weight of the chick. Adult and chick morphometrics were collected using the same protocol as for our study birds, and body condition was calculated using the same model. We omitted one female with an anomalously low residual body condition of −58, which was probably due to measurement error (population mean = −1, s.d. = 10).

### 2.4. Statistical analysis

All statistical analysis was performed in R 4.2.3 [68]. Logistic regressions and generalized linear models (GLMs) were conducted using base R, and mixed-effect models were conducted using the R package ‘lme4’ [69]. We compared hypothesized models using Akaike’s information criterion corrected for sample size (AICc) [70]. A difference in AICc value of 2 identified a better performing model. When the delta AICc value was less than 2, we chose the simpler model.

For predictors of nest attendance (objective one) and differences in attendance rates by sex, we built fractional logistic regressions following Papke & Wooldridge [71]. A strength of this method is that unlike beta-regression, the quasibinomial modelling approach permits values of [0, 1], which were present in our data. Models were weighted by the number of nights of RFID data. We modelled attendance in incubation and chick rearing separately, since hormone samples were collected at different times of day and therefore not comparable. The global models for both sexes during incubation (excluding interactions) included the independent variables bond duration, nest attendance of the mate, body condition, the mate’s body condition, age, PRL, CORT and year, with nest attendance as the dependent variable. The global model for chick rearing excluded year as an independent variable because of the low sample size of chick-rearing pairs in 2019 but was otherwise the same as the global model for incubation in both sexes. Because of the large number of possible predictors and a danger of overfitting, we accepted models with a maximum degrees of freedom (d.f.) of *N*/10 + 1. We noticed a possible three-way interaction between pair experience, PRL and female attendance on male attendance during incubation. To explore this further, we conducted a post hoc analysis where we evaluated the interaction of PRL and female attendance on male attendance for males in experienced and inexperienced pairings separately. We also conducted a post hoc linear model to investigate whether PRL or CORT predicted body condition in incubating females.

For reproductive success (objective 2), we used logistic regression to identify predictors of hatching probability and linear modelling for maximum chick mass. To avoid building a model with an excessive number of parameters, we built model sets for male and female contributions, then used the variables in the best-performing models to build a model set that included the influence of both parents and proceeded with model selection. The global models for the contributions of each parent to hatching success included body condition, PRL, CORT, attendance during incubation, bond duration and age. We added year to the model that combined the independent variables for the lowest AICc models for the contributions of both parents. The global models for chick mass included the same independent variables but we did not add year to the model for the contributions of both parents because of the very different sample sizes between the 2 years. We also compared the likelihood of chicks fledging in each pair experience category in 2019 and 2021 using Fisher’s exact tests.

For our egg volume models using historical data (objective 3), we used categorical pair experience rather than bond duration, because pairs were not selected to decouple mate familiarity from age. However, all eggs were from known-age females, and female age was included as a predictor of egg volume. To identify predictors of egg volume we used a generalized linear mixed model with pair experience, female age, female body condition and female wing cord as independent variables and pair identify as a random effect. To test the effect of egg volume on hatching success, we used a mixed-effects logistic regression with egg volume as the independent variable and pair identity as a random effect. To test the effect of egg volume on maximum chick mass, we used a generalized linear mixed model with egg volume as the independent variable and pair identity as a random effect. Because models for objective 3 had many fewer independent variables than those for objectives 1 and 2, we did not conduct AICc-based model selection and analysed our initial global models.

We checked inflation of variance by multi-collinearity using the variance inflation factors (VIF) for a base model including all variables to be tested (electronic supplementary material, table S6). Hormone measurements were log-adjusted in all models. All prospective models, d.f. and AICc are in electronic supplementary material, tables S2–S5. We calculated pseudo-*R*^2^ values using the Veall–Zimmermann correction [72].

## 3. Results

### 3.1. Objective 1: predictors of nest attendance

During both phases of breeding, there was no evidence for different attendance frequencies in males and females (incubation *p*‐value = 0.14, chick rearing *p*‐value = 0.99; electronic supplementary material, figure S1). Both sexes attended a mean of 59% of nights during incubation and 80% of nights during chick rearing, regardless of pair experience.

**Figure 1.**
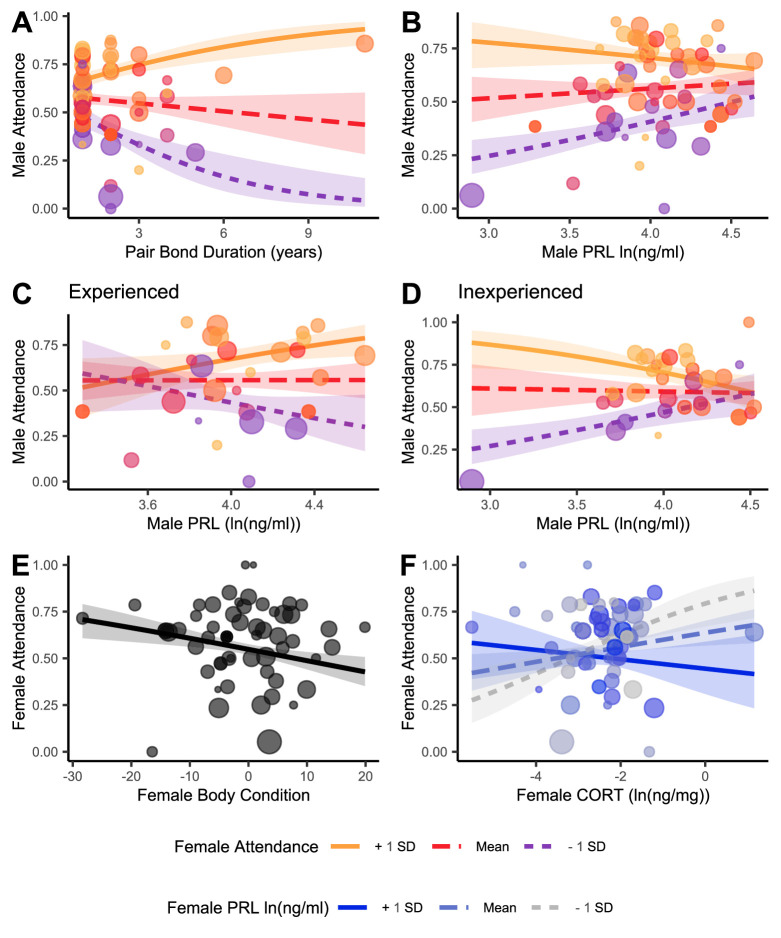
Predictors of incubation nest attendance in males (*a–d*) and females (*e,f*). Shaded areas represent the 95% confidence interval. To show the effects of interactions, results are modelled for the mean values of female attendance (*a–d*) and PRL (*f*), shown in intermediate colours and long dashes, as well as 1 s.d. above and below the mean in solid lines and short dashes, respectively. Hormones are on a log scale. Models were weighted by the number of nights of RFID data, and this is shown by point size. (*a*) Male nest attendance by pair bond duration. (*b*) Male attendance by male PRL. (*c*) Male attendance in experienced pairs by PRL. (*d*) Male attendance in inexperienced pairs by PRL. (*e*) Female attendance by body condition. (*f*) Female attendance by CORT.

The lowest AICc model for male attendance during incubation included a significant interaction between bond duration and female attendance and another significant interaction between female attendance and male PRL as the independent variables (pseudo-*R*^2^ = 0.88; table 1). When they had been together longer, males with an attentive mate were more attentive and males with an inattentive mate were more inattentive (figure 1*a*). Higher PRL was a statistical predictor of greater male attendance, and this effect strengthened with an inattentive mate (figure 1*b*). Post hoc analysis of the response of male attendance to the interaction of female attendance and male PRL revealed contrasting patterns in experienced and inexperienced pairs (experienced pseudo-*R*^2^ = 0.74, inexperienced pseudo-*R*^2^ = 0.92; table 2). In experienced pairs, males with elevated PRL were more attentive when their mate was also attentive but were less attentive when their mate was less attentive (figure 1*c*). In inexperienced pairs, these associations were of a similar magnitude but in the opposite directions (figure 1*d*).

**Table 1.** Logistic regression outputs for incubation and chick-rearing nest attendance rates. All models were weighted by the number of nights of data. Hormone concentrations were log-transformed.

**stage**	**sex**	**predictor**	**estimate**	s.e.	*z*	*p*-value
incubation	M	intercept	−6.27	1.50	−4.19	<0.001
		bond duration	−0.68	0.16	−4.34	<0.001
		female attendance	10.94	2.81	3.89	<0.001
		[PRL]ln(ng ml^−1^)	1.58	0.39	4.04	<0.001
		bond duration ∗ female attendance	1.15	0.25	4.63	<0.001
		[PRL]ln(ng ml^−1^) ∗ female attendance	−2.58	0.72	−3.60	<0.001
	F	intercept	5.23	2.28	2.30	0.022
		male attendance	3.60	0.29	12.29	<0.001
		body condition	−0.02	<0.01	−3.23	0.001
		[PRL]ln(ng ml^−1^)	−1.64	0.56	−2.93	0.003
		[CORT]ln(ng mg^−1^)	2.36	0.89	2.66	0.008
		[PRL]ln(ng ml^−1^) ∗ [CORT]ln(ng mg^−1^)	−0.54	0.22	−2.49	0.013
chick rearing	M	intercept	−0.23	0.76	−0.31	0.757
		female attendance	2.31	0.93	2.49	0.013
	F	intercept	−6.38	1.70	−3.75	<0.001
		male attendance	3.05	0.68	4.48	<0.001
		[prl]ln(ng ml^−1^)	1.45	0.42	3.44	<0.001

**Table 2.** Outcomes of post hoc models of the effects of PRL, female nest attendance and their interaction on male incubation attendance in experienced and inexperienced pairs. Models were weighted by the number of nights of incubation data and PRL was log-transformed.

pair experience	predictor	estimate	s.e.	*z*-value	*p*‐value
experienced	intercept	9.38	5.14	1.82	0.068
	[PRL]ln(ng ml^−1^)	−2.65	1.27	−2.08	0.038
	female attendance	−17.07	8.62	−1.98	0.048
	[PRL]ln(ng ml^−1^) ∗ female attendance	4.94	2.13	2.32	0.020
inexperienced	intercept	−9.24	1.71	−5.41	<0.001
	[PRL]ln(ng ml^−1^)	2.11	0.45	4.64	<0.001
	female attendance	19.46	3.42	5.69	<0.001
	[PRL]ln(ng ml^−1^) ∗ female attendance	−4.34	0.89	−4.88	<0.001

The lowest AICc model for female incubation attendance included male attendance, female body condition and an interaction between female PRL and CORT as the independent variables (pseudo-*R*^2^ = 0.87; table 1). Incubating females in better body condition were less attentive (figure 1*e*). Additionally, females with lower PRL showed an increase in attendance with elevated CORT but a decrease in attendance when PRL was higher (figure 1*f*). Neither PRL nor CORT predicted body condition in incubating females (CORT *p*‐value = 0.423, PRL *p*‐value = 0.430).

**Figure 2.**
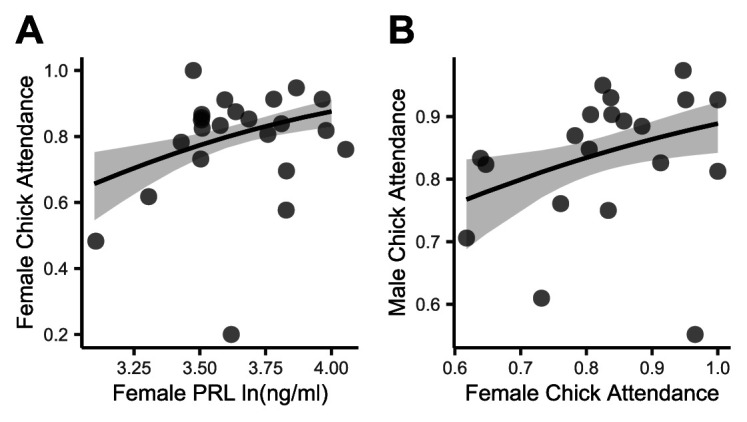
Effects on chick-rearing nest attendance. Shaded areas represent the 95% confidence interval. (*a*) Correlation between plasma PRL (log-transformed) and chick-rearing nest attendance in females. (*b*) Correlation of male and female chick-rearing nest attendance.

The lowest AICc model for male attendance during chick rearing included only female attendance as an independent variable (pseudo-*R*^2^ = 0.28; table 1 and figure 2*b*). The lowest AICc model for females included female PRL and male attendance as independent variables (pseudo-*R*^2^ = 0.64; table 1). Females with higher PRL and more attentive mates were more attentive (figure 2*a*).

### 3.2. Objective 2: predictors of nest success

Eggs were much more likely to hatch in 2021 compared with 2019 (pseudo-*R*^2^ = 0.70; table 3). AICc-based model selection of independent variables for each parent identified female age, bond duration and male attendance as candidate independent variables to predict hatching success, to which we added year to identify the overall predictors of hatching success, including contributions of both parents. Model selection with these variables from both the male and female parent and interactions identified a lowest AICc model with the interaction of pair bond duration and year as the independent variables. Pair bond duration had no effect on hatching success, but with more years of bond duration, pairs benefited less from the change in conditions in 2021 compared with 2019. A GLM of lay date and bond duration showed no correlation (*p* = 0.67), so it is unlikely that differences in measures of reproductive success are due to earlier lay date with mate familiarity.

**Table 3.** Logistic regression and linear model results for measures of reproductive success.

measure of reproductive success	predictor	estimate	s.e.	*z*-value	*p*‐value
hatching success	intercept	−1.61	0.68	−2.36	0.018
	bond duration	0.27	0.22	1.22	0.220
	year(2021)	7.53	2.14	3.52	<0.001
	bond duration ∗ year(2021)	−1.52	0.66	−2.30	0.022
maximum chick weight	intercept	87.05	21.59	4.03	< 0.001
	male attendance	61.82	26.23	2.36	0.025
	female attendance	21.46	22.31	0.96	0.344
	female body condition	−6.56	2.14	−3.07	0.004
	female attendance ∗ female body condition	7.30	2.63	2.78	0.009
chick survival	intercept	3.26	1.02	3.20	0.001
	pair experience (inexperienced)	−2.45	1.10	−2.22	0.027

Only nine chicks hatched in 2019, and only four fledged, compared with 36 chicks in 2021, 33 of which fledged. Fisher’s exact test supported more fledged chicks in experienced compared with inexperienced pairs in 2019 (*p* = 0.048), but no difference in 2021 (*p* = 0.11; electronic supplementary material, figure S2). Because of the very low number of hatched chicks in 2019, we could not perform more sophisticated analyses of year effects. With aggregated years, chicks of experienced pairs were 2.45 times more likely to fledge than those of inexperienced pairs (pseudo-*R*^2^ = 0.70; table 3).

**Figure 3.**
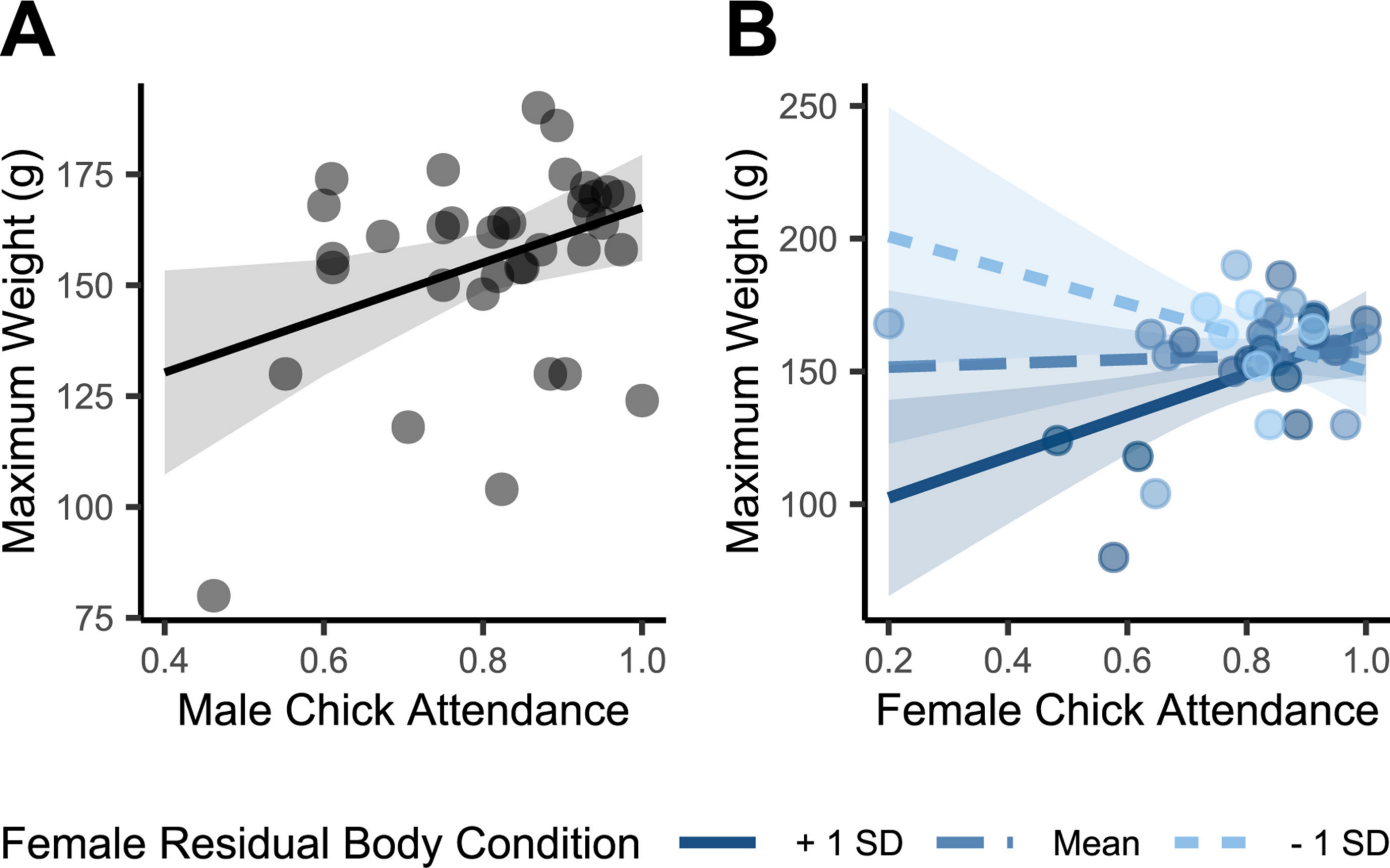
Contributions of males and females to maximum chick weight. The shaded area represents the 95% confidence interval. (*a*) Correlation between the proportion of nights the male was present at the nest and maximum chick mass. (*b*) Contributions of females to maximum chick mass, coloured by three modelled body conditions (mean and ±1 s.d. from the mean) of the female as measured in early incubation.

The lowest AICc model for the contributions of both parents on maximum chick mass included the independent variables male attendance and an interaction between female attendance and female body condition during incubation (electronic supplementary material, table S6). Male attendance during chick rearing predicted greater maximum chick weight (multiple *R*^2^ = 0.39, adjusted *R*^2^ = 0.31; *n* = 36 chicks; table 3 and figure 3*a*), but this was only sometimes the case for females: females that entered the breeding season in better condition showed a more positive effect of nest attendance on chick weight than females in poorer condition (table 3 and figure 3*b*).

### 3.3. Objective 3: egg volume

**Figure 4.**
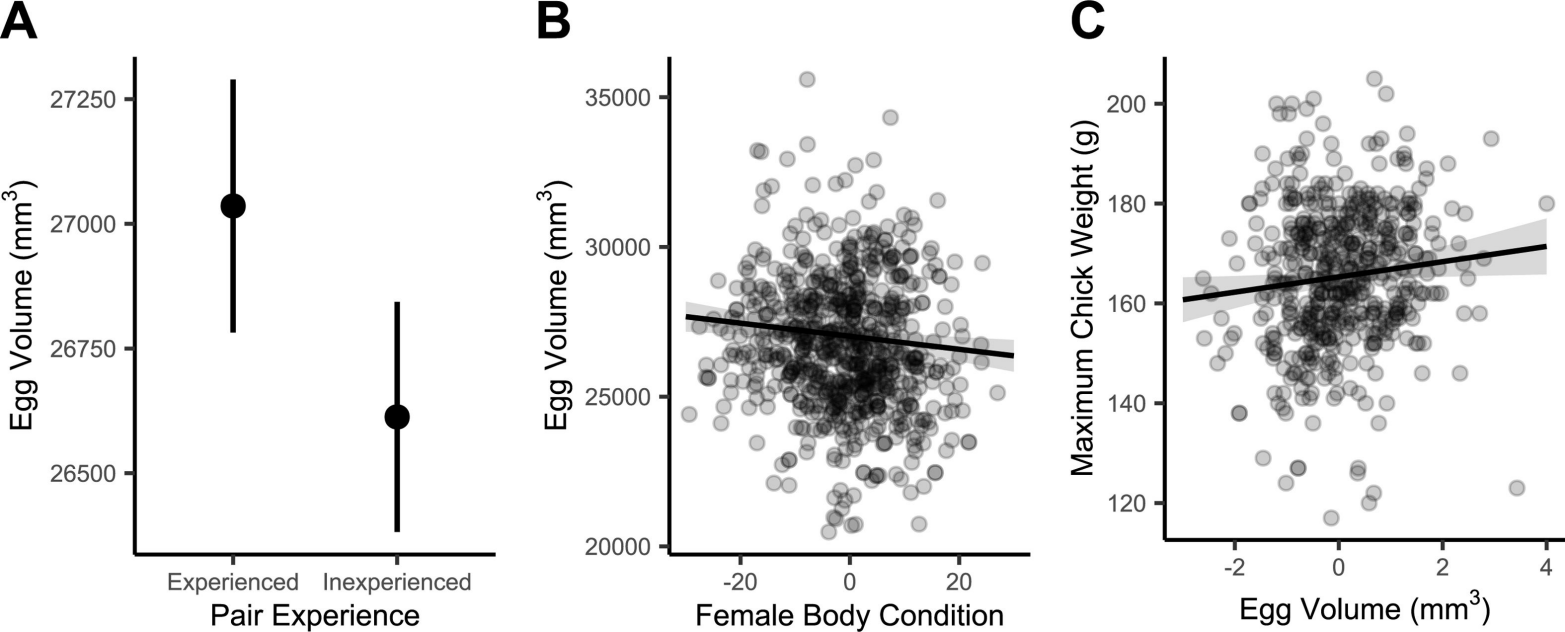
Relationships with egg volume using historical data. The shaded areas represent the 95% confidence interval. (*a*) Difference in egg volume in experienced and inexperienced pairs. (*b*) Correlation between female body condition and egg volume. (*c*) Correlation between egg volume and maximum weight of the resulting chick.

We found that egg volume was greater in experienced pairs than in inexperienced pairs by 423 mm^3^ (s.e. = 137, *t*-value = −3.09, *p* = 0.002; marginal *R*^2^ = 0.02, conditional *R*^2^ = 0.73; figure 4*b*), and females in poorer body condition (measured during incubation, just after lay) had laid larger eggs (estimate = −21.80, s.e. = 7.62, *t*-value = −2.86, *p* = 0.004; figure 4*b*). There was no effect of female age or wing cord on egg volume (age *p*‐value = 0.85, wing cord *p*‐value = 0.15). The model intercept was at 23 359 mm^3^ (s.e. = 2541, *p* < 0.001). There was a non-significant trend towards larger eggs being more likely to hatch (*p* = 0.076; intercept = 1.90, s.e. = 0.24, *p* < 0.001) and larger egg volume was correlated with larger chick maximum weight, although the relationship is noisy and pair identity appears to play an important role (estimate (rescaled) = 1.53, s.e. = 0.70, *t*-value = 2.18, *p* = 0.030, marginal *R*^2^ = 0.01, conditional *R*^2^ = 0.44; figure 4*c*; intercept = 165.31 g, s.e. = 0.75, *p* < 0.001).

## 4. Discussion

The mate familiarity effect is probably a primary benefit of perennial monogamy as a reproductive strategy, with important consequences for the evolution of monogamy. We explored behavioural mechanisms leading to improved reproductive success with mate familiarity in Cassin’s auklet to test the hypothesis that the mate familiarity effect is due to physiologically mediated differences in behaviour with mate familiarity. We found direct evidence of the mate familiarity effect on chick survival to fledging and indirect evidence on maximum chick weight through larger egg volume. Unlike other studies in this and other seabird species [24,41,53,73], we did not find an effect of adult age on any of our fitness or behavioural metrics. Our study was designed to minimize confounds of individual age and pair bond duration, so it is unlikely that the mate familiarity effect we observed is a masked effect of age. Across the 2 years of our study, there were wildly different oceanic conditions. A 2019 was an El Niño year [74], which can have devastating effects on reproductive success in Cassin’s auklet and other seabirds [75–77]. Although hatching and fledging success was lower in 2019 than 2021, we saw no differences in attendance. A possible explanation is an unusual rainstorm that occurred during incubation in 2019, after which many pairs abandoned. It is possible that conditions were sufficient for breeding before the storm, despite the positive ENSO index, and that the rain triggered abandonment. We were not able to replicate the results of others that hatching success improves with mate familiarity [51,52], but we found that experienced pairs suffered a smaller decline in hatching success due to the unfavourable conditions of 2019 than inexperienced pairs.

Although the mate familiarity effect was present, we found partial evidence for our hypothesis in males only. There was an effect of mate familiarity on male behaviour, but no effect in females. Instead, our results reveal important differences between the contributions of males and females, suggesting that the benefits of mate familiarity in Cassin’s auklet may be realized through sex-specific specialization in parenting roles. Sex specialization in parenting is widely demonstrated in the avian literature [78–83], but to our knowledge, ours are the first findings to suggest it is linked to the mate familiarity effect.

*The role of the male:* Only males showed behavioural differences with levels of mate familiarity: the relationship between male and female attendance depended on pair bond duration. When we explored this relationship in experienced and inexperienced pairs separately, our results suggested a central role for PRL in modulating male attendance. The overall correlation between PRL and nest attendance was positive, as in other species [34,84,85], but the strength depended on female behaviour. This result suggests that adjustment of PRL secretion may be a mechanism by which males modulate nest attendance in response to the behaviour of their mate in a pair experience-dependent manner, although an alternative possibility is that increased nest attendance leads to increased prolactin secretion due to increased time spent with the chick.

We found that males more closely matched the attendance of their mate the longer the pair had been together. This could be because better-matched pairs tend to stay together longer, a consequence of better coordination, or males may be modulating their investment in the reproductive attempt based on the likelihood of success. We have never observed a Cassin’s auklet successfully rear offspring after their mate abandons, so they are probably obligately biparental. With more years breeding together, the male could become better attuned to his mate’s behaviour and adjust his attentiveness prudently when her attendance suggests that she may abandon, as has been demonstrated in female black-legged kittiwakes *Rissa tridactyla* with experimentally handicapped mates [86]. Female incubation attendance was correlated with her body condition, which was also an important predictor of the maximum mass attained by the chick. By responding to his mate’s attendance during incubation, the male may be using information that predicts her contributions to chick rearing. While this may not contribute directly to the mate familiarity effect, it would mirror a trade-off between current and future reproduction widely demonstrated in long-lived animals [87–89].

Our endocrine results suggest that PRL may be responsible for this modulation of reproductive attendance, although the negative correlation we found in males with the most inattentive mates is difficult to explain. PRL plays a complex role in physiology, with hundreds of known effects [30,90,91], and it is hypothesized that PRL operates as an intermediary between external stimuli and the expression of parental behaviour rather than on parental behaviour directly [30]. Our result could therefore be due to interactions with other physiological processes. It is also possible that males with longer bond duration require lower levels of PRL to induce parental behaviour as has been suggested in rodents [92]. Experimental manipulation and a more thorough understanding of the neuroendocrinological mechanisms that produce context-dependent relationships between PRL and parental attendance are necessary to understand how PRL could negatively correlate with attendance.

In inexperienced pairs, PRL may play an unprecedented role in sexual conflict and compensation. Theoretical and meta-analytic work suggest that compensation for an underperforming mate plays a critical role in the maintenance of biparental parenting [93], and compensation has indeed been reported in numerous avian species [94–96]. Female Cassin’s auklets have an energetic handicap reflected in the negative correlation between body condition and attendance, so there is reason for male compensation. Supporting this idea, when paired with an inattentive mate, the relationship between PRL and male attendance in inexperienced pairs was positive, which suggests PRL encourages compensation. However, when his mate was more attentive, the relationship between PRL and attendance was negative, suggesting sexual conflict over the negotiation of care. This context-dependent relationship in inexperienced pairs could promote the male either taking advantage of his partner’s attentiveness or compensating for her inattentiveness to avoid nest failure.

*The role of the female*: Rather than responding to external information as in males, female attendance was mostly associated with her internal state and bond duration. The negative correlation between body condition and incubation attendance suggests that females are energetically limited in their parental investment after laying, similar to other seabirds [79,97], since greater nest attendance is associated with declines in body condition and, therefore, a presumed depletion of physiological energy stores. We found larger egg volumes in experienced pairs using a historical dataset, although marginal *R*^2^ was low and conditional *R*^2^ was high, suggesting that pair identity (and thus, individual quality) is important. A larger egg correlated with a heavier chick, agreeing with other work in this species [98]. We also found a negative correlation between body condition post-lay and egg volume, confirming the expense of a larger egg. This appears to negatively impact her ability to provide care: although male and female attendance was equal, female attendance in study birds was only correlated with a larger maximum chick weight when she entered the breeding season in good condition. This is probably due to a difference in the volume of food brought to the chick: despite attending at the same rate, a female in poor condition may consume much of the prey encountered while foraging, rather than bringing it to the chick like a male or female in good condition would. Although we found an interaction of PRL and CORT that affected female incubation attendance, it was not associated with body condition, which is unlike other avian species [85,99,100]. Since how she navigates the trade-off between egg investment and chick care is determined by pair experience, we suggest that the female’s contribution to the mate familiarity effect is in greater investment in the egg, a contribution only she can make, which results in greater reliance on the male to provide high-quality parental care.

## 5. Conclusion

The differences in the drivers of nest attendance between male and female Cassin’s auklets and their contributions to reproductive outcomes suggest that males and females are playing sex-specific roles, as reported in other seabirds [78–80]. We suggest that an evolutionary benefit of perennial monogamy is that the rapport built within the pair enables them to play to their individual strengths resulting from anisogamy. When data from our study birds and historical data are taken together, the results suggest that Cassin’s auklet females are energetically constrained in the parental care they can provide by the high energetic cost of the egg. The female could either lay a smaller, less energetically expensive egg and have a greater ability to compensate for the attendance of the male, should he be inattentive, or she could lay a larger egg that will hatch into a more robust chick at the cost of providing high-quality parental care. Divorce is most common following nest failure [15,101], so if she is breeding with a familiar mate, it is most likely that she has previously raised a chick with him successfully and knows his parenting abilities. Greater egg investment is less risky with a known mate than an unfamiliar one, and this could form the female’s contribution to the benefits of mate familiarity. The contribution of the male is primarily providing parental care, as he does not pay the energetic cost of the egg and we found no evidence that he was limited by body condition. Our results point to improved responsiveness to his mate’s behaviour through a context-dependent action of PRL during incubation, although further work is needed to elucidate mechanisms. It is generally hypothesized that the mate familiarity effect is due to improved coordination [102–104] but sex-specific specialization in parenting roles is another promising driver for the mate familiarity effect.

## Data Availability

Data and code are available in the Dryad repository: [105]. Supplementary material is available online [106].
